# Development and validation of a health profession education-focused scholarly mentorship assessment tool

**DOI:** 10.1007/s40037-018-0491-0

**Published:** 2019-01-10

**Authors:** Christina St-Onge, Meredith Young, Lara Varpio

**Affiliations:** 1 Department of Medicine, Faculty of Medicine and Health Sciences, Université de Sherbrooke, Sherbrooke, Quebec Canada; 2 Department of Medicine, Centre for Medical Education, McGill University, Montreal, Quebec Canada; 3 Department of Medicine, Uniformed Services University of the Health Sciences, Bethesda, MD USA

**Keywords:** Scholarly Mentorship, Innovation, Assessment

## Abstract

**Problem:**

PhD-trained researchers working in health professions education (HPE) regularly engage in one-on-one, or one-on-few, scholarly mentorship activities. While this work is often a formal expectation of these scientists’ roles, rarely is there formal institutional acknowledgement of this mentorship. In fact, there are few official means through which a research scientist can document the frequency or quality of the scholarly mentorship they provide.

**Approach:**

**Outcomes:**

The STHPE assessment tool has appropriate psychometric properties and evidence supporting acceptability. It can be used to document areas of strength and areas for improvement for research scientists engaged in HPE-related scholarly mentorship.

**Next steps:**

At present, the STHPE assessment tool is the only formally developed tool for which there is evidence of validity for use by PhD-trained researchers working in HPE to collect feedback on their scholarly mentorship skills. The STPHE has been used in promotion and tenure packages to document effectiveness and quality of scholarly mentorship.

**Electronic supplementary material:**

The online version of this article (10.1007/s40037-018-0491-0) contains supplementary material, which is available to authorized users.

## Problem

Health professions education scholarship (HPES) is a collaborative endeavour [1] involving scholars across areas of expertise and formal training—including clinician educators [2] who engage in educational activities and PhD-trained HPES research scientists [2] with knowledge of research methods and theories. For HPES research scientists who do not teach in graduate programs (i. e. Master’s and/or PhD programs), many use scholarly mentorship [3] to fulfil their teaching mandate when applying for academic promotion [3]. This mentoring is often explicitly listed in HPES research scientists’ job descriptions as a significant contribution to their *teaching *workload (i. e., scholarly mentorship is often incorporated within the teaching expectations in faculty job descriptions, subsumed along with educational activities such as offering faculty development workshops, classroom instruction, and/or workplace-based teaching) [4]. Unfortunately, while *informally* recognized as teaching, scholarly mentorship is not often *formally* recognized as such by promotion and tenure committees [5].

The gap between what is required of HPES research scientists and what is recognized for promotion and tenure is problematic. Without formal recognition of the educational support offered in these scholarly mentorship collaborations, HPES research scientists are structurally disincentivized from engaging in this work [5]. Recognizing the scholarly mentorship provided by HPE research scientists requires a means for documenting and evaluating this work. In this manuscript, we describe the development of a tool and validation process we applied to establish the appropriateness of (a) documenting the quality of scholarly mentorship provided by HPES research scientists, and (b) providing feedback to the scientists so that they can improve their scholarly mentorship skills.

## Approach

We used DeVellis’s [6] 8‑step framework to develop the tool, and Messick’s unified theory of validity [7, 8] informed our validation approach. In addition, our approach to validation was informed by Van der Vleuten’s [9] utility formula for consideration for the tool acceptability. This study was approved by the research ethics review boards and carried out at three Canadian institutions: McGill University, the Ottawa Hospital, and Université de Sherbrooke.

*Step 1* of DeVellis’s framework is to define the construct of interest. Our construct is the scholarly mentorship provided by HPES research scientists in research collaborations. To ensure compatibility across all three institutions and alignment with how promotion and tenure committees label scholarly mentorship as *teaching*, we chose to use the term *teaching* as defined by the Oxford English Dictionary to label our construct: *“to inform, to train, to give instruction to, to impart knowledge, to show by way, to instruct.”* This definition encompasses the scholarly mentorship activity of interest to us [5], and maps across job descriptions and promotion criteria for HPES research scientists across our three institutions.

We explored the relevant literature and policy documents to identify the skill domains required to engage in scholarly mentorship. Appendix 1, which can be found in the online Electronic Supplementary Material, shows all the references used to generate the list of domains. In addition, we consulted:

- Job descriptions and contracts of the HPES research scientists conducting this study;
- Clinician educators (*n* = 5) who had received scholarly mentorship from one of the team members (see Tab. 1 for the survey questions);
- Informal discussions with senior HPES research scientists (*n* = 2).

**Table 1:** Acceptability survey administered during the development and validation of the Scholarly Mentorship Assessment Tool

Items	*N*	% Agree
Do you think that the assessment form is comprehensive? (i. e. does is address all of the aspects of the one-on-one teaching work that the individual you are evaluating does?)	18	60
Would you be willing to fill out the STHPE assessment form on scientists with which you work?	23	76.7
With respect to language choices, did you think that the assessment form is easy to understand?	22	73.3

Using an inductive analysis approach [10], we identified six domains that constitute the skills required of HPES research scholars when engaging in scholarly mentorship:

A. Expertise in scientific or scholarly field;
B. Expertise in research methodologies, methods, and processes;
C. Proficiency in the development of a feasible, coordinated research plan;
D. Ability to offer support in terms of resources, motivation, and/or professional development;
E. Ability to offer support in terms of networking and increasing visibility; and
F. Ability to support the dissemination and communication of research findings.

In* Step 2*, the domains identified in Step 1 serve as the blueprint for the development of the assessment tool. We generated an item pool by completing the following processes in sequential order:

1. Review published literature to identify relevant surveys and other assessment tools (see Appendix 1).
2. Identify specific items that matched a domain in the blueprint.
3. Collaborative brainstorming with the research team to generate or adapt items for the domains identified and for relevant information to be documented in the assessment tool (for example: number of research meetings, satisfaction with mentorship, structure of the learning activities, etc.). The team members have content expertise (LV, HPES units), and methodological expertise (MY, CSO: assessment and validity; LV: inductive analysis of qualitative data)
4. Review and edit the list of items for clarity and to remove redundancies.

We identified 26 items for the six domains, 8 background items, and 9 satisfaction items, for a total of 41 items.

*Step 3* calls for the selection of a structure for the assessment tool. We created a 4-point Likert Scale for items (Totally disagree, Disagree, Agree, Totally Agree), and included a *not applicable* option to mirror survey examples found in Steps 1 and 2 (Appendix 1).

Aligned with Messick’s conceptualization of evidence of content, we consulted experts and asked them to review the item pool included in the assessment tool (v1) during *Step 4. *Stakeholders included:

1. HPES research scientists (*n* = 5; 3 PhD-trained and 2 MSc-trained) external to the research team;
2. HPES administrative leaders (*n* = 2);
3. HPES research scientist (*n* = 1 PhD-trained who also participated in group 1), and an HPES administrative leader (*n* = 1 MD-trained); and
4. Clinician educators (*n* = 4 MD-trained collaborators).

Via email, we shared the assessment tool (v1) and asked each participant to comment on (1) the clarity of each item, (2) the appropriateness of items, (3) any gaps or missing elements, (4) potential improvements, and (5) the feasibility and acceptability of the tool in their setting. Each stakeholder was consulted independently, and no results were shared across participants.

After replies were received, the researchers met to discuss and implement changes to the assessment tool. By the end of *Step 5*, the assessment tool (v2) consisted of 25 items mapped to the six domains (5 items each for domains A, B and C; 3 items each for domains D and E, 4 items for domain F), six background questions, and two satisfaction questions for a total of 33 items.

This tool was translated into French to facilitate use across Canada. The translation accuracy was confirmed via back-translation [11]. See Appendix 2, in the online Electronic Supplementary Material, for the English version.

## Outcomes

Aligned with Messick’s conceptualization of evidence of structure, in *Step 6* we invited 53 MD collaborators or graduate students (10 from University of Ottawa, 23 from McGill University, 20 from Université de Sherbrooke) to complete the tool. The goal was to be able to establish the psychometric qualities of our tool. During this step, which DeVellis labels as *administering the tool to a development sample*, we also asked participants to complete a survey regarding the tool’s acceptability. To protect anonymity, a research assistant from a different participating university contacted participants (e. g. participants from McGill were contacted by a research assistant from Ottawa). Data were consolidated and anonymized by the research assistant before being returned to the researchers.

Thirty individuals participated, spanning a wide range of experience with HPES projects (*n* = 18 were working on their first HPE project; *n* = 13 had one on-going project; *n* = 10 had 2–3 ongoing projects; *n* = 2 had +10 ongoing projects). The number of HPES projects previously completed varied, ranging from 0 to 25 (mean of 4.9 and SD of 5.6). Participants held a wide range of academic ranks (*n* = 16 were assistant professors; *n* = 6 were associate professors; *n* = 4 were full professors; *n* = 1 medical student; *n* = 6 did not disclose). Multiple disciplines were represented including Anaesthesiology, Cardiology, Emergency Medicine, Family Medicine, Medicine, Paediatrics and Surgery.

We combined DeVellis’s *Steps 7* (evaluation of the items) *and 8* (scale optimization) in analyzing the feedback offered by participants in Step 6. Sociodemographic items were analyzed descriptively. Relying on principles of Classical Test Theory [12], we conducted an item analysis to assess the psychometric properties (item difficulty and discrimination) and internal consistency (Cronbach’s alpha) of the scale. Classical Test Theory was chosen to inform our analysis because it reflects our intended descriptive use of the scores [12].

The scale’s reliability as measured by Cronbach’s alpha was 0.96 for the 25 teaching related items. The difficulty coefficients ranged from 0.88 to 1 with a mean of 0.94 (SD = 0.03). The discrimination coefficients ranged from 0.07 to 0.97, with a mean of 0.70 (SD = 0.24). The psychometric properties of the tool offer evidence of its structure, as defined by Messick. Tab. 1 presents the results of the acceptability survey (collected during Step 6) regarding the STHPE assessment tool. This evidence supports the acceptability of the tool. Together, this evidence supports the appropriateness of the score interpretation in the context of PhD-trained research mentorship in HPE.

## Next Steps

The tool we developed—the Scholarly Teaching in Health Professions Education (STHPE) assessment tool—is an evidence-informed means for enumerating, assessing, and offering feedback on the scholarly mentorship offered by HPES research scientists. Knowing that different HPES stakeholders viewed the scholarly mentorship offered by HPES research scientists in varying ways [5], we developed the STHPE assessment tool through ongoing consultation with multiple stakeholder groups. We suggest that the STHPE has been appropriately vetted for use by HPES research scientists as a means of legitimizing the scholarly mentorship work we do. In fact, the STHPE was successfully used by one team member (MY) who included it in her promotion package and passed promotion and tenure evaluations.

## Caption Electronic Supplementary Material

Relevant litterature used to inform the tool development

Appendix 2
