# Supplementary material for: Development and validation of a health profession education-focused scholarly mentorship assessment tool
Source: Perspect Med Educ. 2019 Jan 10;8(1):43–6. doi: 10.1007/s40037-018-0491-0 (PMC6382618; doi:10.1007/s40037-018-0491-0)
Supplement: Supplementary file 1 — Relevant litterature used to inform the tool development [file 40037_2018_491_MOESM1_ESM.docx]

**Appendix 1 : Literature drawn on for construct definition and tool development**

| Anderson L, Silet K, Fleming M. Evaluating and Giving Feedback to Mentors: New Evidence‐Based Approaches. *Clin Transl Sci. 2012*;*5*(1):71-77. |
| --- |
| Thorndyke LE, Gusic ME, Milner RJ. Functional mentoring: a practical approach with multilevel outcomes. *J Contin Educ Health Prof*. 2008;28(3):157-164. |
| Beecroft PC, Santner S, Lacy ML, Kunzman L, Dorey F. New graduate nurses’ perceptions of mentoring: six‐year programme evaluation. *J Adv Nurs.* 2006;*55*(6):736-747. |
| Feldman MD, Arean PA, Marshall SJ, Lovett M, O’Sullivan P. Does mentoring matter: results from a survey of faculty mentees at a large health sciences university. *Med Educ Online*. 2010;15:5063 |
| Kouzes JM, Posner BZ. *The leadership practices inventory*. San Diego, CA: Pfeiffer & Company; 1988 |
| Walker WO, Kelly PC, Hume JRF. Mentoring for the new millennium. *Med Educ Online*. 2002;7(1):4543. Accessed 15 September 2003 |
| Carlisle C, Calman L, Ibbotson T. Practice-based learning: the role of practice education facilitators in supporting mentors. *Nurse Educ Today*. 2009;29(7):715-721. |
| Hudson PB. Mentors report on their own mentoring practices. *Aust J Teach Educ*. 2010;35(7):30-42. |
| Haggard DL, Dougherty TW, Turban DB, Wilbanks JE. (2011). Who is a mentor? A review of evolving definitions and implications for research. *J Manag*. 2011;37(1):280-304. |
| Sawatzky JAV, Enns CL. (2009). A mentoring needs assessment: Validating mentorship in nursing education. *J Prof Nurs*. 2009;25(3):145-150. |
| Ratnapalan S. (2010). Mentoring in medicine. *Can Fam Phys*. 2010;56(2):198 |
