# Supplementary material for: Development and validation of a health profession education-focused scholarly mentorship assessment tool
Source: Perspect Med Educ. 2019 Jan 10;8(1):43–6. doi: 10.1007/s40037-018-0491-0 (PMC6382618; doi:10.1007/s40037-018-0491-0)
Supplement: Supplementary file 2 — Appendix 2 [file 40037_2018_491_MOESM2_ESM.pdf]

Assessment Form

Scholarly Teaching in Health Professions Education (STHPE)

Many interactions that you have with __________ INSERT NAME __________ are likely not consistent traditional conceptions of university teaching.

For the purpose of this survey, we rely on the definition of "teaching" provided by the Oxford English Dictionary:
"to inform, to train, to give instruction to, to impart knowledge, to show by way, to instruct"

Please refer to this definition as you complete this assessment form.

PART I: SKILLS

Please indicate your level of agreement for each of the following statements, place an X in one box for each statement.

#	Skills	Totally disagree	Somewhat disagree	Somewhat agree	Totally agree	N/A
**A**	**Expertise in scientific or scholarly area(s)**					
A1	Makes complex concepts understandable.	☐	☐	☐	☐	☐
A2	Demonstrates expertise in Health Professions Education research.	☐	☐	☐	☐	☐
A3	Provides referrals to others whose expertise may be relevant to my scholarly area of interest.	☐	☐	☐	☐	☐
A4	Suggests literature (or references) that are pertinent to my areas of interest.	☐	☐	☐	☐	☐
A5	Provides appropriate guidance for methodological choices.	☐	☐	☐	☐	☐
**B**	**Guidance in research and academic skills**					
B1	Helps me formulate educational research question(s).	☐	☐	☐	☐	☐
B2	Helps me with academic writing (e.g., grants, research proposals, ethics submissions, research papers, etc).	☐	☐	☐	☐	☐
B3	Works with me through research-related challenges in a concrete way.	☐	☐	☐	☐	☐
B4	Helps me think of my work more critically / analytically.	☐	☐	☐	☐	☐
B5	Has a positive impact on my approach to research in health professions education.	☐	☐	☐	☐	☐

PART I: SKILLS

#	Skills	Totally disagree	Somewhat disagree	Somewhat agree	Totally agree	N/A
**C**	**Development of a feasible, coordinated research plan**					
C1	Provides valuable feedback on my scholarly work.	☐	☐	☐	☐	☐
C2	Helps me design feasible research projects.	☐	☐	☐	☐	☐
C3	Guides me in conducting feasible research projects.	☐	☐	☐	☐	☐
C4	Helps me design a coordinated program of research.	☐	☐	☐	☐	☐
C5	Motivates me to look for research opportunities in my daily education activities that align with my program of research.	☐	☐	☐	☐	☐
**D**	**Provision of support in terms of : resources, motivation, and professional development**					
D1	Fosters my scholarly independence.	☐	☐	☐	☐	☐
D2	Discusses long-term research goals with me.	☐	☐	☐	☐	☐
D3	Provides me with opportunities to build on prior successes.	☐	☐	☐	☐	☐
**E**	**Provision of support in terms of : networking, visibility, and opportunities**					
E1	Supports me in identifying potential resources to further my scholarly work.	☐	☐	☐	☐	☐
E2	Provides me with networking opportunities that support my scholarly efforts.	☐	☐	☐	☐	☐
E3	Promotes my accomplishments to others.	☐	☐	☐	☐	☐
**F**	**Provision of support in terms of : communicating findings**					
F1	Helps me identify the most appropriate venues (e.g. conferences) for presenting my work.	☐	☐	☐	☐	☐
F2	Helps me prepare for oral presentations and/or posters.	☐	☐	☐	☐	☐
F3	Helps me identify the most appropriate journals for my work.	☐	☐	☐	☐	☐
F4	Helps me prepare and publish my work.	☐	☐	☐	☐	☐

PART II: SATISFACTION

On a scale of 0 to 10, please indicate your level of overall satisfaction in your interactions with

________ INSERT NAME ________ up to now . ☐0 ☐1 ☐2 ☐3 ☐4 ☐5 ☐6 ☐7 ☐8 ☐9 ☐10

(0 = Extremely dissatisfied to10 = Extremely satisfied)

Would you recommend working with ________ INSERT NAME ________ to your colleagues? Yes ☐ No ☐

PART III: BACKGROUND QUESTIONS / SOCIO-DEMOGRAPHIC INFORMATION

1 Please indicate the steps completed to date for the research project you are currently working on with ________ INSERT NAME ________ . [Please indicate all applicable responses by placing an X in the corresponding box(es).]

Step #	**Project Step**	Started	Completed
1	Literature Review	☐	☐
2	Defining the Research Question	☐	☐
3	Writing the Research Protocol	☐	☐
4	Ethics Application	☐	☐
5	Data Collection	☐	☐
6	Data analysis	☐	☐
7	Interpretation of Results	☐	☐
8	Dissemination	☐	☐

2 Is this your first health profession education project with ________ INSERT NAME ________ ? Yes ☐ No ☐ No current project ☐

3 How many projects are currently on-going with ________ INSERT NAME ________ ☐ ?

4 How many previous projects have you undertaken in health profession education? ☐

5 Please provide the following socio-demographic information:

i) Your current academic rank
[Choose only one response by putting an X in the corresponding box.]

Assistant Professor	☐
Associate Professor	☐
Full Professor	☐
Resident	☐
Medical student	☐
Other (please specify): ________	☐

ii) Your clinical affiliation/department/program [________________].

iii) Level of education
[Please indicate all applicable responses by placing an X in the corresponding box(es).]

MD	☐	Specify discipline:
MEd	☐	
MSc/MA	☐	Specify discipline:
PhD	☐	Specify discipline:
Other	☐	Specify:

PART III: BACKGROUND QUESTIONS / SOCIO-DEMOGRAPHIC INFORMATION

6 What aspects of your interactions with ________ INSERT NAME ________ were key to the progress of your scholarly project?

7 Any other general comments

Thank you for taking the time to complete this evaluation.

Version anglaise : Scholarly Teaching in Health Professions Education (STHPE)
Lara Varpio, PhD, Meredith Young, PhD, & Christina St-Onge, PhD
